# Worsened Ability to Engage in Social and Physical Activity During the COVID-19 Pandemic and Older Adults’ Mental Health: Longitudinal Analysis From the Canadian Longitudinal Study on Aging

**DOI:** 10.1093/geroni/igad086

**Published:** 2023-08-19

**Authors:** Theodore D Cosco, Andrew Wister, John R Best, Indira Riadi, Lucy Kervin, Shawna Hopper, Nicole E Basta, Christina Wolfson, Susan A Kirkland, Lauren E Griffith, Jacqueline M McMillani, Parminder Raina

**Affiliations:** Department of Gerontology, Simon Fraser University, Vancouver, British Columbia, Canada; Oxford Institute of Population Ageing, University of Oxford, Oxford, UK; Department of Gerontology, Simon Fraser University, Vancouver, British Columbia, Canada; Department of Gerontology, Simon Fraser University, Vancouver, British Columbia, Canada; Department of Gerontology, Simon Fraser University, Vancouver, British Columbia, Canada; Department of Gerontology, Simon Fraser University, Vancouver, British Columbia, Canada; Department of Gerontology, Simon Fraser University, Vancouver, British Columbia, Canada; Department of Epidemiology, Biostatistics and Occupational Health, McGill University, Montreal, Quebec, Canada; Department of Epidemiology, Biostatistics and Occupational Health, McGill University, Montreal, Quebec, Canada; Research Institute of the McGill University Health Centre, Montreal, Quebec, Canada; Department of Community Health & Epidemiology, and Division of Geriatric Medicine, Faculty of Medicine, Dalhousie University, Halifax, Nova Scotia, Canada; Department of Health Research Methods, Evidence, and Impact, Faculty of Health Sciences, McMaster University, Hamilton, Ontario, Canada; McMaster Institute for Research on Aging, McMaster University, Hamilton, Ontario, Canada; Cumming School of Medicine, Division of Geriatric Medicine, University of Calgary, Alberta, Canada; O’Brien Institute for Public Health, University of Calgary, Alberta, Canada; Department of Health Research Methods, Evidence, and Impact, Faculty of Health Sciences, McMaster University, Hamilton, Ontario, Canada; McMaster Institute for Research on Aging, Labarge Centre for Mobility in Aging, McMaster University, Hamilton, Ontario, Canada

**Keywords:** CLSA, Depression and anxiety, Physical distancing, Social isolation

## Abstract

**Background and Objectives:**

Restrictions implemented to mitigate the transmission of coronavirus disease 2019 (COVID-19) affected older adults’ ability to engage in social and physical activities. We examined mental health outcomes of older adults reporting worsened ability to be socially and physically active during the pandemic.

**Research Design and Methods:**

Using logistic regression, we examined the relationship between positive screen for depression (10-item Center for Epidemiological Studies—Depression Scale) or anxiety (7-item Generalized Anxiety Scale) at the end of 2020 and worsened ability to engage in social and physical activity during the first 6–9 months of the pandemic among older adults in Canada. Interactions between ability to participate in social and physical activity and social participation pre-COVID (2015–2018) and physical activity were also examined. We analyzed data collected before and during the COVID pandemic from the Canadian Longitudinal Study on Aging, a nationally representative longitudinal cohort: pre-pandemic (2015–2018), COVID-Baseline survey (April to May 2020), and COVID-Exit survey (September to December 2020).

**Results:**

Of the 24,108 participants who completed the COVID-Exit survey, 21.96% (*n* = 5,219) screened positively for depression and 5.04% (*n* = 1,132) for anxiety. Worsened ability to participate in social and physical activity was associated with depression (odds ratio [OR] = 1.85 [95% confidence interval {CI} 1.67–2.04]; OR = 2.46 [95% CI 2.25–2.69]), respectively, and anxiety (OR = 1.66 [95% CI 1.37–2.02] and OR = 1.96 [95% CI 1.68–2.30]). Fully adjusted interaction models identified a buffering effect of social participation and the ability to participate in physical activity on depression (*χ*^2^ [1] = 8.86, *p *= .003 for interaction term).

**Discussion and Implications:**

Older adults reporting worsened ability to participate in social and physical activities during the COVID-19 pandemic had poorer mental health outcomes than those whose ability remained the same or improved. These findings highlight the importance of fostering social and physical activity resources to mitigate the negative mental health impacts of future pandemics or other major life stressors that may affect the mental health of older adults.

**Translational Significance:** The coronavirus disease 2019 pandemic has raised concerns regarding the detrimental effect of diminished engagement in physical and social activities for all age groups. We found that older adults who reported worsened ability to engage in physical and social activities as compared to pre-pandemic levels presented greater frequencies of poor mental health outcomes than those whose ability remained the same or improved. These findings underscore a potentially protective influence of social and physical activity against the development of poor mental health outcomes among older adults and the detrimental impact of diminished ability to engage in physical and social activities.

## Background and Objectives

The escalating incidence of mental illness has garnered increased attention during the coronavirus disease 2019 (COVID-19) pandemic (Holmes et al., 2020). Prior to the pandemic, an estimated one in five Canadians experience mental illness in any given year (Blazer et al., 2012), with depression and anxiety accounting for a Canadian economic burden of approximately $32 and $17 billion annually, respectively (Lim et al., 2008; Smetanin et al., 2015). Worsening health outcomes and mental health status resulting from the pandemic may be particularly deleterious for older adults who experience social and material deprivation (Marmot, 2005).

Restrictions including physical distancing, social isolation, and stay-at-home orders have been used as strategies to combat viral transmission, often challenging the ability of individuals to maintain social connections and physical activity (Aubertin-Leheudre & Rolland, 2020; Tison et al., 2020). This enforced disengagement is of concern given the well-established associations between social and physical activity levels and the development, duration, and severity of depressive and anxiety disorders (McDowell et al., 2019; Seo Jin & Chao, 2018; Vink et al., 2008). In keeping with theories of reserve in the context of mental health and resilience, it has been suggested that older adults that have greater resources, such as social connectedness and physical health, may better respond to adversity compared to individuals with lower reserves (Cosco et al., 2018). Recent research has found a positive association between physical activity levels and older adults’ mental well-being during the COVID-19 pandemic (Callow et al., 2020; Carriedo et al., 2020). Similarly, a study by Hu and colleagues (2021) found that face-to-face inter-household socialization during the COVID-19 pandemic was associated with better mental health. A recent longitudinal study by Ang (2022) found that the association between social support and depressive symptoms weakened during the pandemic, suggesting that the imposed restrictions may have buffered the protective effects of social support on older adults’ mental health.

Despite emerging research examining the association between physical and social activity and older adults’ mental health during the COVID-19 pandemic, there is a lack of research focused on the Canadian population (though see Raina et al., 2021). As the experience of the COVID-19 pandemic, and the resulting restrictions that were implemented, differ between countries, it is important to understand how the COVID-19 pandemic affected Canadians specifically. Using longitudinal data from the Canadian Longitudinal Study on Aging (CLSA), we aimed to examine the association between the ability to engage in social and physical activity with changes in depression and anxiety during the initial wave of the COVID-19 pandemic between April and December 2020, in a Canadian sample of mid-life and older adults. As associations between changes in behavior with changes in depression and anxiety might depend in part on pre-pandemic levels of these behaviors (Blumenthal et al., 1999), our secondary aim was to assess interactions between pre-COVID levels of social participation and physical activity and pandemic-related worsened ability to engage in physical and social activity, in relation to older adults’ mental health. It was hypothesized that individuals who perceived that their ability to engage in social and physical activities was negatively affected by COVID-19 restrictions, would show greater increases in depression and anxiety, compared to individuals who reported that their activities were not affected. Additionally, it was hypothesized that the gap would be larger for individuals with lower pre-COVID levels of social participation and physical activity.

## Research Design and Methods

### Study Design and Participants

The CLSA is a large (*n* = 51,338), nationally representative longitudinal cohort of Canadian residents aged 45–85 at CLSA Baseline (2011–2015). Study participants are followed every 3 years for at least 20 years or until death or loss of follow-up. Questionnaires are completed by the full sample; a subset of 30,997 participants who live within 25–50 km of one of the 11 Data Collection Sites across seven provinces are interviewed in their homes and visit a Data Collection Site to complete physical assessments and to provide biological samples. In mid-2018, the first CLSA follow-up data collection was completed (Follow-up 1, *n* = 48,893; 95% retention), which has been used as the “Pre-COVID” period in these analyses. Detailed design and methods for the CLSA are described elsewhere (Raina et al., 2019).

Launched on April 15, 2020, the CLSA COVID-19 questionnaire study aims to better understand the epidemiology of COVID-19 and the social and mental health implications of the pandemic on the lives of older Canadian residents. Between April 15 and May 30, 2020, COVID-Baseline data were collected via an initial 30-min web-based questionnaire that was either completed directly online by the participant or data were entered for the participants by trained CLSA interviewers eliciting the information over the telephone. Between September 29 and December 29, 2020, a 30-min exit questionnaire was completed (COVID-Exit).

Of the full (*n* = 51,338) CLSA sample, 42,700 study participants were invited to participate in the CLSA COVID-19 study. Excluded participants consisted of those who had died (*n* = 2,500), withdrew from the study (*n* = 3,406), required a proxy to participate in the study (*n* = 318), or were not invited for other administrative reasons, such as outdated contact information (*n* = 2,414). Participants were recruited either by email (*n* = 34,498), if they had an active email address, or by telephone (*n* = 8,202). Information on the scope and purpose of the study was provided to prospective participants before consent to participate was obtained. The recruitment process revealed additional participants who were deceased (*n* = 166) or in need of a proxy to participate (*n* = 23). The final eligible sample was thus 42,511, of whom 67.18% (*n* = 28,559) agreed to participate in the COVID-Baseline, and of whom 84.41% (*n* = 24,114) participated in COVID-Exit.

The analyses presented here use data from the CLSA Follow-up 1 (i.e., Pre-COVID), COVID-Baseline, and COVID-Exit to model the relationship between physical and social activity and mental health. The analytic sample size for the current study was limited to participants that participated in the COVID-Exit survey; because missing data differed across the two outcome measures, the analytic sample size was 23,765 for the depression outcome and 22,446 for the anxiety outcome. The CLSA and CLSA COVID studies were approved by research ethics boards at all the participating research institutions across Canada.

### Measurement of Study Variables

#### Outcomes

Assessing depressive symptoms in the past week, the 10-item Center for Epidemiological Studies—Depression Scale (CES-D-10) includes two positive affect items alongside eight depressed affect items, with response categories 0–3: rarely or never (<1 a day); some of the time (1–2 days); occasionally (3–4 days); all of the time (5–7 days; Andresen et al., 1994). Positive items were reversed, and participant scores were summed, resulting in scores ranging from 0 to 30, with higher scores indicating greater depressive symptomatology. A cutoff score of 10 was used to dichotomize into positive (scores 10–30) and negative (scores 0–9) screens for depression (Andresen et al., 1994). The CES-D-10 was administered at Pre-COVID, COVID-Baseline, and COVID-Exit.

Identifying probable cases of generalized anxiety disorder (GAD), the 7-item Generalized Anxiety Scale (GAD-7; Spitzer et al., 2006) includes items developed to reflect the *Diagnostic and Statistical Manual of Mental Disorders, Fourth Edition (DSM-IV)* symptom criteria. Study participants are asked to respond to the presence of anxiety symptoms in the last 2 weeks with 0: not at all; 1: several days; 2: more than half the days; 3: nearly every day. Scores range from 0 to 21, with higher scores indicating greater anxiety symptoms and a score of 10 or more indicating a positive screen for anxiety. GAD-7 scores were dichotomized into positive (scores 10–21) and negative (scores 0–9) screens for anxiety. The GAD-7 was administered at COVID-Baseline and COVID-Exit.

#### Exposures

At COVID-Exit, participants were asked how their perceived functional ability and daily activities had changed since March 1, 2020, with the following prompts: “Your ability to engage in physical activity (walking, exercise, working out) has become …”; “Your ability to participate in the community and maintain a social life (e.g., volunteer, connect with others) has become….” Participants could respond with: “Much better”; “A little better”; “Same”; “A little worse”; “Much worse.” Responses were dichotomized into worse (“Much worse” and “A little worse”) and same/better (“Same,” “A little better,” and “Much better”).

#### Covariates

Due to concern that the association between the exposures and mental health during COVID might be confounded by demographic, behavioral, and pre-COVID mental health, models were adjusted for age group (<55, 55–64, 65–74, ≥75), dwelling type (house: including single detached, semi-detached, duplex or townhouse; apartment or condominium; other: senior’s housing, mobile home, and hotel), geographic area (urban, rural), household composition (living alone, not living alone), and smoking status (never smoker, former smoker, current smoker) at COVID-Baseline; alcohol consumption (did not drink in past 12 months including participants who never drink, occasional drinker, binge drinker, regular drinker) at COVID-Exit; and multimorbidity (number of chronic conditions: 0, 1, 2, ≥3), physical activity (high vs low), total annual household income (<$20,000, $20,000–$49,999, $50,000–$99,999 $100,000–$149,999, ≥$150,000), and social participation (high vs low) at Pre-COVID. Chronic conditions were counted if they were among the following disease categories: musculoskeletal, respiratory, cardiovascular, endocrine-metabolic, neurological, gastrointestinal, genitourinary, ophthalmologic, renal, and cancer (Supplementary Table 1)

#### Moderators

Both moderator variables were collected during the pre-COVID period. The Physical Activity Scale for the Elderly (PASE) was dichotomized into high versus low based on the World Health Organization’s (WHO) threshold of physical activity of at least 150 min of moderate-intensity or at least 75 min of vigorous-intensity physical activity per week (Washburn et al., 1999). Social participation was dichotomized into high versus low at the median of the summed frequency of age-group adjusted (<55, 55–64, 65–74, ≥75) participation in eight activity categories participated in the month previous to data collection (Dozois, 2020): sports, clubs, outdoors, neighborhood, volunteer, church, other activities. Response categories for these activities ranged from “never” (coded as 0) to “at least once a day” (coded as 10). Age adjustment was done to account for expected differences in activity frequency across the middle and older adulthood.

### Statistical Analysis

Binary logistic regression was used to examine the relationship between exposures (worse vs same/better ability to participate in physical and social activity) and outcomes (positive vs negative screens for depression and anxiety). Models were fit initially without covariates, followed by adjustment for the covariates described earlier. Additionally, models were adjusted for baseline COVID levels of depression, CES-D-10 at Pre-COVID and COVID-Baseline, GAD-7 screen at COVID-Baseline and whether participants had ever been diagnosed with a mood disorder or anxiety disorder Pre-COVID. Interactions between the ability to participate in social and physical activity and Pre-COVID social participation and PASE scores were examined in adjusted logistic regression models. These primary analyses involved listwise deletion of missing data on covariates, exposures, and outcomes.

Analyses of missing data indicated nonrandom missingness for outcome (Supplementary Table 2) and exposure (Supplementary Table 3) variables. Younger participants and males were more likely to have missing exposure and outcome variables. Further, participants with positive depression and anxiety screens at COVID-Baseline were more likely to have missing outcomes and exposures at COVID-Exit. To address potential bias introduced by systematic missingness at COVID-Exit, multiple imputation by chained equations was used to impute missing data across all outcomes, exposures, and covariates. Forty imputed data sets were created using the R package *mice.* After confirming the convergence of the samplings algorithm using trace plots, results were pooled across these data sets using the Barnard–Rubin procedure to estimate pooled standard errors and degrees of freedom (Barnard & Rubin, 1999; Rubin, 1987).

Primary analyses were conducted using Stata (version 17.0), and multiple imputation and figures were created using R Software version 4.1.0 (r-project.org).

## Results

In COVID-Exit, 21.96% (*n* = 5,219) of participants had a positive screen for depression and 5.04% (*n* = 1,132) had a positive screen for anxiety. Among participants who had a positive screen for depression, 78.61% (*n* = 4,014) reported worsened ability to participate in social activity and 43.93% (*n* = 2,268) in physical activity. Among participants who had a positive screen for anxiety, 81.41% (*n* = 898) reported worsened ability to participate in social activity and 47.42% (*n* = 2,268) in physical activity (Table 1).

**Table 1.** Comparison of Descriptive Statistics by Depression and Anxiety Screen at COVID-Exit

Characteristic	Positive depression screen		Positive anxiety screen	
	No *n* = 18,546	Yes *n* = 5,219	No *n* = 21,314	Yes *n* = 1,132
Baseline COVID age group, in years				
<55	777 (4.19%)	316 (6.05%)	929 (4.36%)	110 (9.72%)
55–64	5,537 (29.86%)	1,682 (32.23%)	6,445 (30.24%)	473 (41.78%)
65–74	6,911 (37.26%)	1,785 (34.20%)	7,860 (36.88%)	348 (30.74%)
>75	5,321 (28.69%)	1,436 (27.51%)	6,080 (28.53%)	201 (17.76%)
Sex				
Female	9,311 (50.20%)	3,318 (63.58%)	11,049 (51.84%)	741 (65.46%)
Male	9,235 (49.80%)	1,901 (36.42%)	10,265 (48.16%)	391 (33.54%)
Dwelling type				
House	14,729 (79.51%)	3,761 (72.19%)	16,657 (78.24%)	857 (75.84%)
Apartment	3,161 (17.06%)	1,198 (22.99%)	3,851 (18.09%)	238 (21.06%)
Senior’s housing	382 (2.06%)	156 (2.99%)	477 (2.24%)	21 (1.86%)
Institution	32 (0.17%)	14 (0.27%)	39 (0.18%)	4 (0.35%)
Hotel	108 (0.58%)	51 (0.98%)	142 (0.67%)	7 (0.62%)
Other	113 (0.61%)	30 (0.58%)	124 (0.58%)	3 (0.27%)
(Missing)	21	9	24	2
Urban living (versus rural)	16,038 (86.95%)	4,681 (90.05%)	18,539 (87.44%)	1,015 (89.98%)
(Missing)	100	21	111	4
Tobacco cigarette use				
Not at all	17,364 (94.53%)	4,739 (91.84%)	19,910 (94.23%)	990 (88.79%)
Occasionally	212 (1.15%)	83 (1.61%)	252 (1.19%)	25 (2.24%)
Daily	793 (4.32%)	338 (6.55%)	968 (4.58%)	100 (8.97%)
(Missing)	177	59	184	17
Alcohol use				
Never	3,230 (17.46%)	1,064 (20.43%)	3,850 (18.10%)	243 (21.52%)
Less than once a month	2,067 (11.17%)	662 (12.71%)	2,388 (11.23%)	156 (13.82%)
About once a month	1,261 (6.82%)	345 (6.62%)	1,437 (6.76%)	75 (6.64%)
2–3 times a month	1,850 (10.00%)	505 (9.70%)	2,090 (9.82%)	109 (9.65%)
Once a week	1,823 (9.85%)	435 (8.35%)	2,055 (9.66%)	87 (7.71%)
2–3 times a week	3,338 (18.04%)	808 (15.51%)	3,764 (17.69%)	159 (14.08%)
4-5 times a week	2,037 (11.01%)	572 (10.98%)	2,334 (10.97%)	127 (11.25%)
Almost every day	2,895 (15.65%)	817 (15.69%)	3,355 (15.77%)	173 (15.32%)
(Missing)	45	11	41	3
Lives alone				
Do not live alone	14,020 (76.96%)	3,462 (67.80%)	15,748 (75.15%)	822 (74.05%)
Live alone	4,198 (23.04%)	1,644 (32.20%)	5,208 (24.85%)	288 (25.95%)
(Missing)	328	113	358	22
Annual income ($CAD)				
<20,000	570 (3.26%)	264 (5.44%)	718 (3.6%)	63 (6.02%)
20,000–49,999	3,523 (20.16%)	1,242 (25.61%)	4,180 (20.83%)	247 (23.59%)
50,000–99,999	6,663 (38.13%)	1,805 (37.22%)	7,595 (37.85%)	367 (35.05%)
100,000–149,999	3,662 (20.95%)	878 (18.11%)	4,135 (20.61%)	215 (20.53%)
150,000+	3,058 (17.50%)	660 (13.61%)	3,437 (17.13%)	155 (14.80%)
(Missing)	1,070	370	1,249	85
Multimorbidity				
0	2,651 (14.68%)	528 (10.38%)	2,927 (14.10%)	115 (10.39%)
1	4,075 (22.57%)	847 (16.65%)	4,510 (21.73%)	199 (17.98%)
2	4,003 (22.17%)	1,042 (20.48%)	4,569 (22.01%)	223 (20.14%)
3+	7,328 (40.58%)	2,671 (52.50%)	8,751 (42.16%)	570 (51.49%)
(Missing)	489	131	557	25
Pre-COVID physical activity				
Below WHO threshold	12,058 (65.41%)	3,748 (72.36%)	14,093 (66.50%)	800 (71.56%)
Above WHO threshold	6,356 (34.59%)	1,432 (27.64%)	7,100 (33.50%)	318 (28.44%)
(Missing)	112	39	122	14
Pre-COVID social participation				
Low	9,553 (52.43%)	3,046 (59.71%)	11,251 (53.74%)	662 (59.96%)
High	8,669 (47.57%)	2,055 (40.29%)	9,685 (46.26%)	442 (40.04%)
(Missing)	324	118	378	28
Social participation ability during COVID				
Same/better	6,292 (34.48%)	1,092 (21.39%)	6,822 (32.47%)	205 (18.59%)
Worse	11,957 (65.52%)	4,014 (78.61%)	14,190 (67.53%)	898 (81.41%)
(Missing)	297	113	302	29
Physical activity ability during COVID				
Same/better	14,734 (80.08%)	2,895 (56.07%)	16,175 (76.47%)	592 (52.58%)
Worse	3,665 (19.92%)	2,268 (43.93%)	4,977 (23.53%)	534 (47.42%)
(Missing)	147	56	162	6
Pre-COVID mood disorder	2,266 (12.42%)	1,704 (33.35%)	3,238 (15.44%)	472 (42.68%)
(Missing)	301	109	343	26
Pre-COVID anxiety disorder	1,127 (6.18%)	938 (18.34%)	1,598 (7.62%)	318 (28.86%)
(Missing)	299	105	341	30
Baseline COVID anxiety screen, positive	403 (2.32%)	907 (19.34%)	791 (3.93%)	449 (42.48%)
(Missing)	1,171	530	1,204	75
Baseline COVID depression screen, positive	1,837 (10.05%)	2,945 (57.67%)	3,624 (17.24%)	779 (70.18%)
(Missing)	266	112	294	22

*Notes*: COVID = coronavirus; WHO = World Health Organization.

Ability to participate in social activity during the COVID-19 pandemic was perceived to be worse for 68.31% (*n* = 16,063; Supplementary Table 4). Ability to participate in physical activity was perceived to be worse for 25.19% (*n* = 5,976) of participants (Supplementary Table 4).

The results of the two primary logistic regression models, one using a positive screen for depression as the outcome, and one using a positive screen for anxiety as the outcome, are presented in Table 2. Unadjusted odds ratios (ORs) for the associations between the exposures and outcomes were consistently larger than the covariate-adjusted ORs, which adjust for demographic confounders and baseline COVID mental health (see Table 2). The estimated ORs and confidence intervals (CIs) were very similar when analyzing the complete case data set and when analyzing the following imputation of missing data (Table 2, bottom). Here we focus on the covariate-adjusted effects from the complete case data. Participants who reported worsened ability to participate in social activity were more likely to have a positive screen at COVID-Exit for depression (OR = 1.85 [95% CI 1.67–2.04]) as well as anxiety (OR = 1.66 [95% CI 1.37–2.02]), compared to those whose ability was reported to be the same or better. Participants who reported worsened ability to participate in physical activity were more likely to have a positive screen at COVID-Exit for depression (OR = 2.46 [95% CI 2.25–2.69]) as well as anxiety (OR = 1.96 [95% CI 1.68–2.30]) compared to those whose ability was reported to be the same or better.

**Table 2.** Summary of Primary Logistic Regression Models

Exposure	Positive depression screen			Positive anxiety screen		
	Odds ratio	95% CI	*p* Value	Odds ratio	95% CI	*p* Value
Analysis of complete case data						
Social participation ability during COVID						
Same/better	1 (ref)			1 (ref)		
Worse (unadjusted)	1.93	1.80–2.08	<.001	2.11	1.80–2.48	<.001
Worse (adjusted)	1.85	1.67–2.04	<.001	1.66	1.37–2.02	<.001
Physical activity ability during COVID						
Same/better	1 (ref)			1 (ref)		
Worse (unadjusted)	3.15	2.95–3.36	<.001	2.93	2.60–3.31	<.001
Worse (adjusted)	2.46	2.25–2.69	<.001	1.96	1.68–2.30	<.001
Analysis of multiply-imputed data						
Social participation ability during COVID						
Same/better	1 (ref)			1 (ref)		
Worse (Unadjusted)	1.92	1.79–2.08	<.001	2.04	1.75–2.38	<.001
Worse (Adjusted)	1.75	1.61–1.92	<.001	1.61	1.35–1.92	<.001
Physical activity ability during COVID						
Same/better	1 (ref)			1 (ref)		
Worse (Unadjusted)	3.13	2.94–3.33	<.001	2.94	2.56–3.33	<.001
Worse (Adjusted)	2.50	2.33–2.70	<.001	1.96	1.69–1.27	<.001

*Notes*: CI = confidence interval; COVID = coronavirus. Odds ratios are adjusted for age group, sex, dwelling type, urban versus rural setting, tobacco cigarette use, alcohol consumption, living alone versus with others, annual income, multimorbidity, pre-COVID depression and anxiety, and baseline COVID depression and anxiety screen.

**Figure 1.**
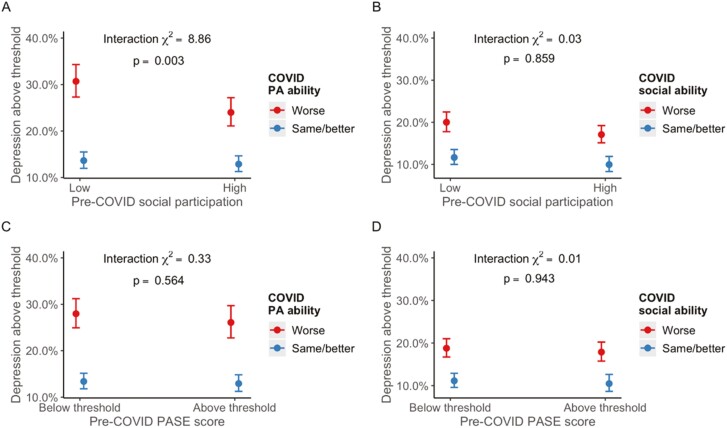
Plots of marginal effects from interaction logistic regression models with depression as outcome. COVID = coronavirus; PA = physical ability; PASE = The Physical Activity Scale for the Elderly.

**Figure 2.**
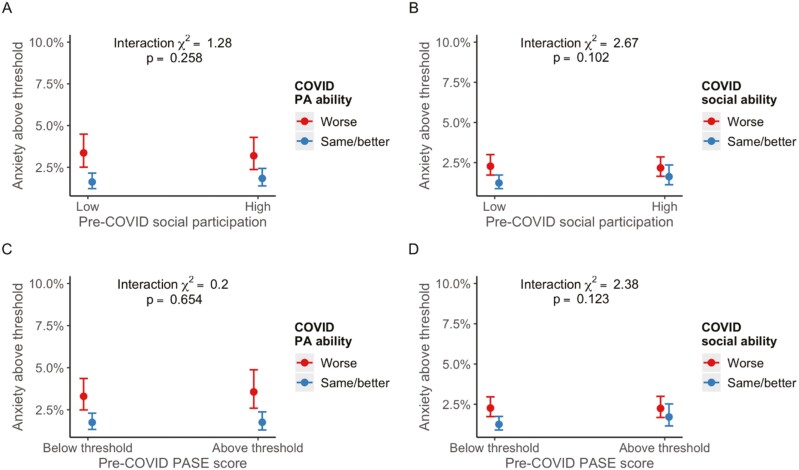
Plots of marginal effects from interaction logistic regression models with anxiety as outcome. COVID = coronavirus; PA = physical ability; PASE = The Physical Activity Scale for the Elderly.

Fully adjusted interaction models identified a buffering effect of social participation and ability to participate in physical activity on depression (χ^2^ [1] = 8.86, *p *= .003 for interaction; see Figure 1A). Simple effects analysis revealed that the odds of depression for those who experienced worsened ability to engage in physical activity was stronger among those who had a low level of pre-COVID social participation (OR = 2.81, 95% CI 2.49–3.17) as compared to those with a high level of pre-COVID social participation (OR = 2.14, 95% CI 1.87–2.44). No other interactions were observed for the outcomes of depression (Figure 1B–D) and anxiety (Figure 2A–D).

The primary analyses were repeated using nondichotomized values of the mental health outcomes, pre-COVID exposures, and COVID-era perceived effects on social and physical activity. As shown in Supplementary Table 5, in both unadjusted and adjusted models, we observed that worsened ability to engage in physical and social activities was associated with higher depression and anxiety scores. The results from the interaction models are shown in Supplementary Figures 1 and 2 and suggest a similar pattern of results presented earlier using dichotomized variables.

## Discussion and Interpretation

Using longitudinal data from the CLSA, a large national sample of community-dwelling older adults, we observed an association between worsened ability to participate in social and physical activity and greater frequency of positive screens for depression and anxiety during the first 9 months of the COVID-19 pandemic. Although these relationships were attenuated after adjustment for demographic, socioeconomic, and health covariates, including baseline COVID mental health, persistent links between worsened ability to engage in social and physical activities and poor mental health were observed. Further, we observed that the association between worsened ability to participate in physical activity and the odds of a positive depression screen was attenuated among those with high levels of Pre-COVID social participation, suggesting a potential buffering effect of this variable.

These results may support the hypothesis that individuals who perceived that their social and physical activities were negatively affected by COVID-19 restrictions, such as physical distancing and lockdowns, were at increased risk of poor mental health (i.e., positive screen for depression and/or anxiety) compared to populations of individuals who reported that their activities were not impacted. Associations between participation in social as well as physical activities and better mental health among older adults are well established (Ansseau et al., 2008; Vink et al., 2008; Wang et al., 2018); however, the potential impact of lower social and physical activity that has occurred during government-imposed restrictions has not been studied in a Canadian context. The introduction of an external force-limiting social and physical activity presents a unique circumstance, with implications for the timing, duration, and limitations of future mitigation strategies to limit viral transmission.

The buffering effect of Pre-COVID social participation suggests that greater pre-pandemic social capital may have some impact in offsetting negative pandemic-related mental health sequelae. Previous research has established that social participation is directly related to mental health outcomes in older adults outside the context of the COVID-19 pandemic (Thoits, 2011), and there is some evidence that social participation might buffer against the negative effects of experienced pain on depression (Ang & Chen, 2019). The current findings suggest that social participation may play a moderating role within the context of the COVID-19 pandemic to blunt some of the negative mental health impacts associated with required changes in behavior. The precise mechanism is unclear but could involve social participation, providing a substitute for the diminishment of physical activity. Fostering greater social resources may be a mechanism by which the negative implications of reduced capacity to engage in social and physical activities can be offset.

The longitudinal nature of the CLSA and the timing of data collection relative to the enforcement of Canadian lockdown restrictions adds strength to this study. Participants completed COVID-Baseline depression and generalized anxiety screens within 2 months of the WHO declaring COVID-19 a global pandemic. COVID-Exit depression and generalized anxiety screens were collected after stricter restrictions were put in place, limiting social and physical activity. We were able to adjust for lifetime history of mood or anxiety disorder diagnoses, 2015-2018 CES-D-10 scores in the Pre-COVID wave, in addition to including CES-D-10 scores at COVID-Baseline in modeling COVID-Exit CES-D-10 screens for depression. Further, in modeling COVID-Exit GAD-7 screens, we were able to adjust for lifetime history of mood or anxiety disorder diagnoses, and COVID-Baseline GAD-7 scores. After the inclusion of these baseline COVID mental health variables in regression models, although attenuated, associations between depression and anxiety and worsened ability to participate in social and physical activity the magnitude of the ORs remained over 2.0, which speaks to the robustness of these relationships.

There are several limitations that must be considered in the interpretation of these results. Foremost, our outcome measures are screening tools, rather than diagnostic tools; therefore, we are unable to determine whether the exposure variables affect the actual presence of depression or generalized anxiety. Although the CLSA is a longitudinal study, the exposure and outcome variables were measured at the same time (COVID-Exit) using subjective self-report questionnaires. There are limitations to the conclusions that can be extrapolated from these data. In adjusting for COVID-Baseline depression and anxiety levels, we were only able to adjust for CES-D-10 scores in models of CES-D-10 and GAD-7 at COVID-Exit; GAD-7 scores were not available at COVID-Baseline. The subjectivity of individuals’ self-reported ability to participate in activities and reported levels of depression and anxiety are subject to social desirability bias, which may have resulted in overestimation of the level of social and physical activity as well as underestimation of depression and anxiety (Adams et al., 2005). Additionally, the reason for a change in participants’ ability to engage in social and physical activities cannot be assumed to be due to the COVID-19 pandemic and may be due to other circumstances (i.e., declining health). Further, as detailed in Table 1, the sample is predominately urban dwellers, with the majority of participants not living alone. These characteristics may limit the generalizability of the results as many Canadian older adults will have experienced the COVID-19 pandemic in a rural area with less access to resources and social opportunities and/or lived alone during the imposed restrictions. Similarly, the CLSA sampling frame did not include older adults living in long-term care facilities at recruitment, which may have resulted in the overestimation of the physical and mental health of the sample. Moreover, the generalizability of these findings should be limited to community-dwelling populations.

Intervention studies comparing aspects of functional and structural social support as well as different exercise modalities would be useful in identifying the specific attributes of these behaviors that are driving the relationship with mental health. Further, it would be prudent to identify the root causes of participants’ perceptions of worsened ability to participate in social and physical activity. If, for example, there are financial barriers to participation, this may be a potential avenue for policy intervention via subsidies. If these specific attributes can be identified, it may be possible to integrate these components into future pandemic-related policies to facilitate positive mental health strategies.

## Supplementary Material

igad086_suppl_Supplementary_Material

## Data Availability

Data are available from the CLSA (www.clsa-elcv.ca) for researchers who meet the criteria for access to de-identified CLSA data.
