# Supplementary material for: Worsened Ability to Engage in Social and Physical Activity During the COVID-19 Pandemic and Older Adults’ Mental Health: Longitudinal Analysis From the Canadian Longitudinal Study on Aging
Source: Innov Aging. 2023 Aug 19;7(7):igad086. doi: 10.1093/geroni/igad086 (PMC10533203; doi:10.1093/geroni/igad086)
Supplement: igad086_suppl_Supplementary_Material [file igad086_suppl_supplementary_material.docx]

**Online Supplementary Material**

Supplementary Table 1. Comparison of descriptive statistics by depression and anxiety screen at COVID exit.

| **Characteristic** | **Positive depression screen** | | **Positive anxiety screen** | |
| --- | --- | --- | --- | --- |
|  | No,  n = 18,546 | Yes,  n = 5,219 | No,  n = 21,314 | Yes,  n = 1,132 |
| Baseline COVID age group, in years |  |  |  |  |
| <55 | 777 (4.19%) | 316 (6.05%) | 929 (4.36%) | 110 (9.72%) |
| 55-64 | 5,537 (29.86%) | 1,682 (32.23%) | 6,445 (30.24%) | 473 (41.78%) |
| 65-74 | 6,911 (37.26%) | 1,785 (34.20%) | 7,860 (36.88%) | 348 (30.74%) |
| >75 | 5,321 (28.69%) | 1,436 (27.51%) | 6,080 (28.53%) | 201 (17.76%) |
| Sex |  |  |  |  |
| Female | 9,311 (50.20%) | 3,318 (63.58%) | 11,049 (51.84%) | 741 (65.46%) |
| Male | 9,235 (49.80%) | 1,901 (36.42%) | 10,265 (48.16%) | 391 (33.54%) |
| Dwelling type |  |  |  |  |
| House | 14,729 (79.51%) | 3,761 (72.19%) | 16,657 (7824%) | 857 (75.84%) |
| Apartment | 3,161 (17.06%) | 1,198 (22.99%) | 3,851 (18.09%) | 238 (21.06%) |
| Senior's housing | 382 (2.06%) | 156 (2.99%) | 477 (2.24%) | 21 (1.86%) |
| Institution | 32 (0.17%) | 14 (0.27%) | 39 (0.18%) | 4 (0.35%) |
| Hotel | 108 (0.58%) | 51 (0.98%) | 142 (0.67%) | 7 (0.62%) |
| Other | 113 (0.61%) | 30 (0.58%) | 124 (0.58%) | 3 (0.27%) |
| (Missing) | 21 | 9 | 24 | 2 |
| Urban living (versus rural) | 16,038 (86.95%) | 4,681 (90.05%) | 18,539 (87.44%) | 1,015 (89.98%) |
| (Missing) | 100 | 21 | 111 | 4 |
| Tobacco cigarette use |  |  |  |  |
| Not at all | 17,364 (94.53%) | 4,739 (91.84%) | 19,910 (94.23%) | 990 (88.79%) |
| Occasionally | 212 (1.15%) | 83 (1.61%) | 252 (1.19%) | 25 (2.24%) |
| Daily | 793 (4.32%) | 338 (6.55%) | 968 (4.58%) | 100 (8.97%) |
| (Missing) | 177 | 59 | 184 | 17 |
| Alcohol use |  |  |  |  |
| Never | 3,230 (17.46%) | 1,064 (20.43%) | 3,850 (18.10%) | 243 (21.52%) |
| Less than once a month | 2,067 (11.17%) | 662 (12.71%) | 2,388 (11.23%) | 156 (13.82%) |
| About once a month | 1,261 (6.82%) | 345 (6.62%) | 1,437 (6.76%) | 75 (6.64%) |
| 2-3 times a month | 1,850 (10.00%) | 505 (9.70%) | 2,090 (9.82%) | 109 (9.65%) |
| Once a week | 1,823 (9.85%) | 435 (8.35%) | 2,055 (9.66%) | 87 (7.71%) |
| 2-3 times a week | 3,338 (18.04%) | 808 (15.51%) | 3,764 (17.69%) | 159 (14.08%) |
| 4-5 times a week | 2,037 (11.01%) | 572 (10.98%) | 2,334 (10.97%) | 127 (11.25%) |
| Almost every day | 2,895 (15.65%) | 817 (15.69%) | 3,355 (15.77%) | 173 (15.32%) |
| (Missing) | 45 | 11 | 41 | 3 |
| Lives alone |  |  |  |  |
| Do not live alone | 14,020 (76.96%) | 3,462 (67.80%) | 15,748 (75.15%) | 822 (74.05%) |
| Live alone | 4,198 (23.04%) | 1,644 (32.20%) | 5,208 (24.85%) | 288 (25.95%) |
| (Missing) | 328 | 113 | 358 | 22 |
| Annual income ($CAD) |  |  |  |  |
| <$20K | 570 (3.26%) | 264 (5.44%) | 718 (3.6%) | 63 (6.02%) |
| $20-50K | 3,523 (20.16%) | 1,242 (25.61%) | 4,180 (20.83%) | 247 (23.59%) |
| $50-100K | 6,663 (38.13%) | 1,805 (37.22%) | 7,595 (37.85%) | 367 (35.05%) |
| $100-150K | 3,662 (20.95%) | 878 (18.11%) | 4,135 (20.61%) | 215 (20.53%) |
| 150K+ | 3,058 (17.50%) | 660 (13.61%) | 3,437 (17.13%) | 155 (14.80%) |
| (Missing) | 1,070 | 370 | 1,249 | 85 |
| Multimorbidity |  |  |  |  |
| 0 | 2,651 (14.68%) | 528 (10.38%) | 2,927 (14.10%) | 115 (10.39%) |
| 1 | 4,075 (22.57%) | 847 (16.65%) | 4,510 (21.73%) | 199 (17.98%) |
| 2 | 4,003 (22.17%) | 1,042 (20.48%) | 4,569 (22.01%) | 223 (20.14%) |
| 3+ | 7,328 (40.58%) | 2,671 (52.50%) | 8,751 (42.16%) | 570 (51.49%) |
| (Missing) | 489 | 131 | 557 | 25 |
| Pre-COVID physical activity |  |  |  |  |
| Below WHO threshold | 12,058 (65.41%) | 3,748 (72.36%) | 14,093 (66.50%) | 800 (71.56%) |
| Above WHO threshold | 6,356 (34.59%) | 1,432 (27.64%) | 7,100 (33.50%) | 318 (28.44%) |
| (Missing) | 112 | 39 | 122 | 14 |
| Pre-COVID social participation |  |  |  |  |
| Low | 9,553 (52.43%) | 3,046 (59.71%) | 11,251 (53.74%) | 662 (59.96%) |
| High | 8,669 (47.57%) | 2,055 (40.29%) | 9,685 (46.26%) | 442 (40.04%) |
| (Missing) | 324 | 118 | 378 | 28 |
| Social participation ability during COVID |  |  |  |  |
| Same/better | 6,292 (34.48%) | 1,092 (21.39%) | 6,822 (32.47%) | 205 (18.59%) |
| Worse | 11,957 (65.52%) | 4,014 (78.61%) | 14,190 (67.53%) | 898 (81.41%) |
| (Missing) | 297 | 113 | 302 | 29 |
| Physical activity ability during COVID |  |  |  |  |
| Same/better | 14,734 (80.08%) | 2,895 (56.07%) | 16,175 (76.47%) | 592 (52.58%) |
| Worse | 3,665 (19.92%) | 2,268 (43.93%) | 4,977 (23.53%) | 534 (47.42%) |
| (Missing) | 147 | 56 | 162 | 6 |
| Pre-COVID mood disorder | 2,266 (12.42%) | 1,704 (33.35%) | 3,238 (15.44%) | 472 (42.68%) |
| (Missing) | 301 | 109 | 343 | 26 |
| Pre-COVID anxiety disorder | 1,127 (6.18%) | 938 (18.34%) | 1,598 (7.62%) | 318 (28.86%) |
| (Missing) | 299 | 105 | 341 | 30 |
| Baseline COVID anxiety screen, positive | 403 (2.32%) | 907 (19.34%) | 791 (3.93%) | 449 (42.48%) |
| (Missing) | 1,171 | 530 | 1,204 | 75 |
| Baseline COVID depression screen, positive | 1,837 (10.05%) | 2,945 (57.67%) | 3,624 (17.24%) | 779 (70.18%) |
| (Missing) | 266 | 112 | 294 | 22 |

*Notes*. COVID = coronavirus; WHO = World Health Organization.

Supplementary Table 2. Comparison of descriptive statistics by ability to participate in social and physical activities at COVID exit.

| **Characteristic** | Social participation ability during COVID | | Physical activity ability during COVID | |
| --- | --- | --- | --- | --- |
|  | Worse,  n = 16,063 | Same/better,  n = 7,451 | Worse,  n = 5,976 | Same/better,  n = 17,752 |
| Baseline COVID age group, in years |  |  |  |  |
| <55 | 784 (4.88%) | 294 (3.95%) | 250 (4.18%) | 836 (4.71%) |
| 55-64 | 5,116 (31.85%) | 2,018 (27.08%) | 1,668 (27.91%) | 5,519 (31.09%) |
| 65-74 | 5,932 (36.93%) | 2,674 (35.89%) | 2,132 (35.68%) | 6,563 (36.97%) |
| >75 | 4,231 (26.34%) | 2,465 (33.08%) | 1,926 (32.23%) | 4,834 (27.23%) |
| Sex |  |  |  |  |
| Female | 8,988 (55.95%) | 3,501 (46.99%) | 3,454 (57.80%) | 9,148 (51.53%) |
| Male | 7,075 (44.05%) | 3,950 (53.01%) | 2,522 (42.20%) | 8,604 (48.47%) |
| Dwelling type |  |  |  |  |
| House | 12,693 (79.11%) | 5,610 (75.39%) | 4,348 (72.81%) | 14,113 (79.61%) |
| Apartment | 2,875 (17.92%) | 1,427 (19.18%) | 1,349 (22.59%) | 3,004 (16.94%) |
| Senior's housing | 294 (1.83%) | 240 (3.23%) | 179 (3.00%) | 357 (2.01%) |
| Institution | 18 (0.11%) | 27 (0.36%) | 14 (0.23%) | 32 (0.18%) |
| Hotel | 84 (0.52%) | 75 (1.01%) | 45 (0.75%) | 113 (0.64%) |
| Other | 81 (0.50%) | 62 (0.83%) | 37 (0.62%) | 109 (0.61%) |
| (Missing) | 18 | 10 | 4 | 24 |
| Urban living (versus rural) | 14,128 (88.44%) | 6,378 (85.98%) | 5,396 (90.73%) | 15,296 (86.63%) |
| (Missing) | 88 | 33 | 29 | 95 |
| Tobacco cigarette use |  |  |  |  |
| Not at all | 15,067 (94.60%) | 6,824 (92.64%) | 5,579 (94.27%) | 16,504 (93.87%) |
| Occasionally | 184 (1.16%) | 108 (1.47%) | 71 (1.20%) | 224 (1.27%) |
| Daily | 676 (4.24%) | 434 (5.89%) | 268 (4.53%) | 854 (4.86%) |
| (Missing) | 136 | 85 | 58 | 170 |
| Alcohol use |  |  |  |  |
| Never | 2,684 (16.75%) | 1,544 (20.77%) | 1,113 (18.67%) | 3,163 (17.86%) |
| Less than once a month | 1,832 (11.43%) | 865 (11.64%) | 780 (13.09%) | 1,951 (11.02%) |
| About once a month | 1,087 (6.78%) | 497 (6.69%) | 415 (6.96%) | 1,185 (6.69%) |
| 2-3 times a month | 1,629 (10.17%) | 713 (9.59%) | 569 (9.55%) | 1,791 (10.11%) |
| Once a week | 1,514 (9.45%) | 718 (9.66%) | 538 (9.03%) | 1,715 (9.68%) |
| 2-3 times a week | 2,873 (17.93%) | 1,234 (16.60%) | 984 (16.61%) | 3,151 (17.79%) |
| 4-5 times a week | 1,837 (11.47%) | 748 (10.06%) | 647 (10.85%) | 1,956 (11.04%) |
| Almost every day | 2,566 (16.02%) | 1,114 (14.99%) | 915 (15.35%) | 2,799 (15.80%) |
| (Missing) | 41 | 18 | 15 | 41 |
| Lives alone |  |  |  |  |
| Do not live alone | 12,045 (76.21%) | 5,248 (72.13%) | 4,138 (70.89%) | 13,303 (76.25%) |
| Live alone | 3,761 (23.79%) | 2,028 (27.87%) | 1,699 (29.11%) | 4,144 (23.75%) |
| (Missing) | 257 | 175 | 139 | 305 |
| Annual income ($CAD) |  |  |  |  |
| <$20K | 481 (3.18%) | 348 (4.99%) | 252 (4.52%) | 587 (3.51%) |
| $20-50K | 2,944 (19.47%) | 1,774 (25.46%) | 1,249 (22.41%) | 3,509 (21.00%) |
| $50-100K | 5,704 (37.72%) | 2,652 (38.06%) | 2,137 (38.34%) | 6,318 (37.81%) |
| $100-150K | 3,232 (21.38%) | 1,268 (18.20%) | 1,080 (19.38%) | 3,451 (20.65%) |
| 150K+ | 2,759 (18.25%) | 926 (13.29%) | 856 (15.36%) | 2,847 (17.04%) |
| (Missing) | 943 | 483 | 402 | 1,040 |
| Multimorbidity |  |  |  |  |
| 0 | 2,155 (13.77%) | 991 (13.67%) | 608 (10.36%) | 2,559 (14.84%) |
| 1 | 3,268 (20.88%) | 1,619 (22.33%) | 912 (15.54%) | 4,019 (23.31%) |
| 2 | 3,470 (22.17%) | 1,520 (20.96%) | 1,211 (20.64%) | 3,824 (22.17%) |
| 3+ | 6,760 (43.19%) | 3,121 (43.04%) | 3,136 (53.45%) | 6,843 (39.68%) |
| (Missing) | 410 | 200 | 109 | 507 |
| Pre-COVID physical activity |  |  |  |  |
| Below WHO threshold | 10,438 (65.35%) | 5,186 (70.14%) | 4,029 (67.70%) | 11,753 (66.68%) |
| Above WHO threshold | 5,535 (34.65%) | 2,208 (29.86%) | 1,922 (32.30%) | 5,873 (33.32%) |
| (Missing) | 91 | 57 | 25 | 126 |
| Pre-COVID social participation |  |  |  |  |
| Low | 7,849 (49.74%) | 4,565 (62.52%) | 3,109 (53.04%) | 9,481 (54.41%) |
| High | 7,931 (50.26%) | 2,737 (37.48%) | 2,753 (46.96%) | 7,945 (45.59%) |
| (Missing) | 283 | 149 | 114 | 326 |
| Social participation ability during COVID |  |  |  |  |
| Same/better | -- | -- | 4,858 (82.41%) | 11,162 (63.62%) |
| Worse | -- | -- | 1,037 (17.59%) | 6,383 (36.38%) |
| (Missing) | -- | -- | 81 | 207 |
| Physical activity ability during COVID |  |  |  |  |
| Same/better | 4,858 (30.32%) | 1,037 (13.98%) | -- | -- |
| Worse | 11,162 (69.68%) | 6,383 (86.02%) | -- | -- |
| (Missing) | 43 | 31 | -- | -- |
| Pre-COVID mood disorder | 2,845 (18.00%) | 1,081 (14.79%) | 1,344 (22.86%) | 2,619 (15.02%) |
| (Missing) | 261 | 141 | 96 | 310 |
| Pre-COVID anxiety disorder | 1,430 (9.05%) | 597 (8.17%) | 689 (11.71%) | 1,366 (7.83%) |
| (Missing) | 259 | 140 | 91 | 312 |
| Baseline COVID anxiety screen, positive | 982 (6.57%) | 309 (4.48%) | 529 (9.72%) | 773 (4.67%) |
| (Missing) | 1,121 | 557 | 533 | 1,191 |
| Baseline COVID depression screen, positive | 3,625 (22.90%) | 1,097 (15.00%) | 1,866 (31.85%) | 2,904 (16.61%) |
| (Missing) | 236 | 138 | 117 | 269 |

*Notes*. COVID = coronavirus; WHO = World Health Organization.

Supplementary Table 3. List of assessed comorbidities.

| - Arthritis (including at least one of the following: osteoarthritis of the hand, hip, or knee; rheumatoid arthritis; or other arthritis - Heard disease including congestive heart failure - Peripheral vascular disease - Alzheimer’s or dementia - Multiple sclerosis - Epilepsy - Ulcers - Bowel disorder - Macular degeneration - Cancer - Back problems - Kidney disease or failure - Diabetes - High blood pressure - Under-active thyroid - Over-active thyroid - Angina - Stroke - Heart attack or myocardial infarction - Transient ischemic attack - Asthma - Osteoporosis - Parkinson’s - COPD |
| --- |

Supplementary Table 4. Comparison of demographic characteristics between individuals with and without depression and anxiety outcome variables.

| **Characteristic** | **COVID exit depression screen available** | | **COVID exit anxiety screen available** | |
| --- | --- | --- | --- | --- |
|  | No  n = 4,800 | Yes  n = 23,765 | No  n = 6,119 | Yes  n = 22,446 |
| Baseline COVID age group, in years |  |  |  |  |
| <55 | 425 (8·85%) | 1,093 (4·60%) | 479 (7·83%) | 1,039 (4·63%) |
| 55-64 | 1,729 (36·02%) | 7,219 (30·38%) | 2,030 (33·18%) | 6,918 (30·82%) |
| 65-74 | 1,400 (29·17%) | 8,696 (36·59%) | 1,888 (30·85%) | 8,208 (36·57%) |
| >75 | 1,246 (25·96%) | 6,757 (28·43%) | 1,722 (28·14%) | 6,281 (27·98%) |
| Sex |  |  |  |  |
| Female | 2,357 (49·10%) | 12,629 (53·14%) | 3,196 (52·23%) | 11,790 (52·53%) |
| Male | 2,443 (50·90%) | 11,136 (46·86%) | 2,923 (47·77%) | 10,656 (47·47%) |
| Dwelling type |  |  |  |  |
| House | 3,711 (77·67%) | 18,490 (77·90%) | 4,687 (76·92%) | 17,514 (78·12%) |
| Apartment | 842 (17·62%) | 4,359 (18·37%) | 1,112 (18·25%) | 4,089 (18·24%) |
| Senior's housing | 123 (2·57%) | 538 (2·27%) | 163 (2·68%) | 498 (2·22%) |
| Institution | 17 (0·36%) | 46 (0·19%) | 20 (0·33%) | 43 (0·19%) |
| Hotel | 45 (0·94%) | 159 (0·67%) | 55 (0·90%) | 149 (0·66%) |
| Other | 40 (0·84%) | 143 (0·60%) | 56 (0·92%) | 127 (0·57%) |
| (Missing) | 22 | 30 | 26 | 26 |
| Urban living (versus rural) | 4,162 (87·20%) | 20,719 (87·63%) | 5,327 (87·53%) | 19,554 (87·56%) |
| (Missing) | 27 | 121 | 33 | 115 |
| Tobacco cigarette use |  |  |  |  |
| Not at all | 4,037 (91·73%) | 22,103 (93·94%) | 5,240 (92·17%) | 20,900 (93·95%) |
| Occasionally | 63 (1·43%) | 295 (1·25%) | 81 (1·42%) | 277 (1·25%) |
| Daily | 301 (6·84%) | 1,131 (4·81%) | 364 (6·40%) | 1,068 (4·80%) |
| (Missing) | 399 | 236 | 434 | 201 |
| Alcohol use |  |  |  |  |
| Never | 56 (25·57%) | 4,294 (18·11%) | 257 (16·84%) | 4,093 (18·27%) |
| Less than once a month | 27 (12·33%) | 2,729 (11·51%) | 212 (13·89%) | 2,544 (11·36%) |
| About once a month | 13 (5·94%) | 1,606 (6·77%) | 107 (7·01%) | 1,512 (6·75%) |
| 2-3 times a month | 25 (11·42%) | 2,355 (9·93%) | 181 (11·86%) | 2,199 (9·82%) |
| Once a week | 24 (10·96%) | 2,258 (9·52%) | 140 (9·17%) | 2,142 (9·56%) |
| 2-3 times a week | 26 (11·87%) | 4,146 (17·49%) | 249 (16·32%) | 3,923 (17·51%) |
| 4-5 times a week | 19 (8·68%) | 2,609 (11·00%) | 167 (10·94%) | 2,461 (10·99%) |
| Almost every day | 29 (13·24%) | 3,712 (15·66%) | 213 (13·96%) | 3,528 (15·75%) |
| (Missing) | 4,581 | 56 | 4,593 | 44 |
| Lives alone |  |  |  |  |
| Do not live alone | 3,582 (77·73%) | 17,482 (74·95%) | 4,494 (76·61%) | 16,570 (75·09%) |
| Live alone | 1,026 (22·27%) | 5,842 (25·05%) | 1,372 (23·39%) | 5,496 (24·91%) |
| (Missing) | 192 | 441 | 253 | 380 |
| Annual income ($CAD) |  |  |  |  |
| <$20K | 194 (4·37%) | 834 (3·74%) | 247 (4·37%) | 781 (3·70%) |
| $20-50K | 945 (21·31%) | 4,765 (21·34%) | 1,283 (22·72%) | 4,427 (20·97%) |
| $50-100K | 1,549 (34·93%) | 8,468 (37·93%) | 2,055 (36·38%) | 7,962 (37·71%) |
| $100-150K | 854 (19·26%) | 4,540 (20·34%) | 1,044 (18·48%) | 4,350 (20·60%) |
| 150K+ | 893 (20·14%) | 3,718 (16·65%) | 1,019 (18·04%) | 3,592 (17·01%) |
| (Missing) | 365 | 1,440 | 471 | 1,334 |
| Multimorbidity |  |  |  |  |
| 0 | 711 (15·26%) | 3,179 (13·74%) | 848 (14·28%) | 3,042 (13·91%) |
| 1 | 1,008 (21·64%) | 4,922 (21·27%) | 1,221 (20·56%) | 4,709 (21·54%) |
| 2 | 944 (20·27%) | 5,045 (21·80%) | 1,197 (20·15%) | 4,792 (21·92%) |
| 3+ | 1,995 (42·83%) | 9,999 (43·20%) | 2,673 (45·01%) | 9,321 (42·63%) |
| (Missing) | 142 | 620 | 180 | 582 |
| Pre-COVID physical activity |  |  |  |  |
| Below WHO threshold | 3,198 (67·47%) | 15,806 (66·93%) | 4,111 (68·02%) | 14,893 (66·75%) |
| Above WHO threshold | 1,542 (32·53%) | 7,808 (33·07%) | 1,933 (31·98%) | 7,417 (33·25%) |
| (Missing) | 60 | 151 | 75 | 136 |
| Pre-COVID social participation |  |  |  |  |
| Low | 2,519 (53·92%) | 12,599 (54·02%) | 3,205 (53·82%) | 11,913 (54·05%) |
| High | 2,153 (46·08%) | 10,724 (45·98%) | 2,750 (46·18%) | 10,127 (45·95%) |
| (Missing) | 128 | 442 | 164 | 406 |
| Social participation ability during COVID |  |  |  |  |
| Same/better | 92 (57·86%) | 15,971 (68·38%) | 975 (69·69%) | 15,088 (68·23%) |
| Worse | 67 (42·14%) | 7,384 (31·62%) | 424 (30·31%) | 7,027 (31·77%) |
| (Missing) | 4,641 | 410 | 4,720 | 331 |
| Physical activity ability during COVID |  |  |  |  |
| Same/better | 43 (25·90%) | 5,933 (25·18%) | 465 (32·07%) | 5,511 (24·74%) |
| Worse | 123 (74·10%) | 17,629 (74·82%) | 985 (67·93%) | 16,767 (75·26%) |
| (Missing) | 4,634 | 203 | 4,669 | 168 |
| Pre-COVID mood disorder | 871 (18·70%) | 3,970 (17·00%) | 1,131 (19·05%) | 3,710 (16·80%) |
| (Missing) | 142 | 410 | 183 | 369 |
| Pre-COVID anxiety disorder | 438 (9·40%) | 2,065 (8·84%) | 587 (9·87%) | 1,916 (8·68%) |
| (Missing) | 140 | 404 | 173 | 371 |
| Baseline COVID anxiety screen, positive | 267 (7·00%) | 1,310 (5·94%) | 337 (7·15%) | 1,240 (5·86%) |
| (Missing) | 985 | 1,701 | 1,407 | 1,279 |
| Baseline COVID depression screen, positive | 1,056 (24·96%) | 4,782 (20·45%) | 1,435 (26·15%) | 4,403 (19·90%) |
| (Missing) | 569 | 378 | 631 | 316 |

*Notes*. COVID = coronavirus; WHO = World Health Organization.

Supplementary Table 5. Comparison of demographic characteristics between individuals with and without exposure variables.

| **Characteristic** | **COVID exit PA ability available** | | **COVID exit social ability available** | |
| --- | --- | --- | --- | --- |
|  | No  n = 4,837 | Yes  n = 23,728 | No  n = 5,051 | Yes  n = 23,514 |
| Baseline COVID age group, in years |  |  |  |  |
| <55 | 432 (8·93%) | 1,086 (4·58%) | 440 (8·71%) | 1,078 (4·58%) |
| 55-64 | 1,761 (36·41%) | 7,187 (30·29%) | 1,814 (35·91%) | 7,134 (30·34%) |
| 65-74 | 1,401 (28·96%) | 8,695 (36·64%) | 1,490 (29·50%) | 8,606 (36·60%) |
| >75 | 1,243 (25·70%) | 6,760 (28·49%) | 1,307 (25·88%) | 6,696 (28·48%) |
| Sex |  |  |  |  |
| Female | 2,384 (49·29%) | 12,602 (53·11%) | 2,497 (49·44%) | 12,489 (53·11%) |
| Male | 2,453 (50·71%) | 11,126 (46·89%) | 2,554 (50·56%) | 11,025 (46·89%) |
| Dwelling type |  |  |  |  |
| House | 3,740 (77·71%) | 18,461 (77·89%) | 3,898 (77·54%) | 18,303 (77·93%) |
| Apartment | 848 (17·62%) | 4,353 (18·37%) | 899 (17·88%) | 4,302 (18·32%) |
| Senior's housing | 125 (2·60%) | 536 (2·26%) | 127 (2·53%) | 534 (2·27%) |
| Institution | 17 (0·35%) | 46 (0·19%) | 18 (0·36%) | 45 (0·19%) |
| Hotel | 46 (0·96%) | 158 (0·67%) | 45 (0·90%) | 159 (0·68%) |
| Other | 37 (0·77%) | 146 (0·62%) | 40 (0·80%) | 143 (0·61%) |
| (Missing) | 24 | 28 | 24 | 28 |
| Urban living (versus rural) | 4,189 (87·04%) | 20,692 (87·66%) | 4,375 (87·08%) | 20,506 (87·66%) |
| (Missing) | 24 | 124 | 27 | 121 |
| Tobacco cigarette use |  |  |  |  |
| Not at all | 4,057 (91·58%) | 22,083 (93·97%) | 4,249 (91·63%) | 21,891 (93·98%) |
| Occasionally | 63 (1·42%) | 295 (1·26%) | 66 (1·42%) | 292 (1·25%) |
| Daily | 310 (7·00%) | 1,122 (4·77%) | 322 (6·94%) | 1,110 (4·77%) |
| (Missing) | 407 | 228 | 414 | 221 |
| Alcohol use |  |  |  |  |
| Never | 74 (28·91%) | 4,276 (18·06%) | 122 (25·79%) | 4,228 (18·03%) |
| Less than once a month | 25 (9·77%) | 2,731 (11·54%) | 59 (12·47%) | 2,697 (11·50%) |
| About once a month | 19 (7·42%) | 1,600 (6·76%) | 35 (7·40%) | 1,584 (6·75%) |
| 2-3 times a month | 20 (7·81%) | 2,360 (9·97%) | 38 (8·03%) | 2,342 (9·99%) |
| Once a week | 29 (11·33%) | 2,253 (9·52%) | 50 (10·57%) | 2,232 (9·52%) |
| 2-3 times a week | 37 (14·45%) | 4,135 (17·47%) | 65 (13·74%) | 4,107 (17·51%) |
| 4-5 times a week | 25 (9·77%) | 2,603 (11·00%) | 43 (9·09%) | 2,585 (11·02%) |
| Almost every day | 27 (10·55%) | 3,714 (15·69%) | 61 (12·90%) | 3,680 (15·69%) |
| (Missing) | 4,581 | 56 | 4,578 | 59 |
| Lives alone |  |  |  |  |
| Do not live alone | 3,623 (77·95%) | 17,441 (74·91%) | 3,771 (77·75%) | 17,293 (74·92%) |
| Live alone | 1,025 (22·05%) | 5,843 (25·09%) | 1,079 (22·25%) | 5,789 (25·08%) |
| (Missing) | 189 | 444 | 201 | 432 |
| Annual income ($CAD) |  |  |  |  |
| <$20K | 189 (4·22%) | 839 (3·76%) | 199 (4·26%) | 829 (3·75%) |
| $20-50K | 952 (21·28%) | 4,758 (21·35%) | 992 (21·23%) | 4,718 (21·36%) |
| $50-100K | 1,562 (34·91%) | 8,455 (37·94%) | 1,661 (35·55%) | 8,356 (37·83%) |
| $100-150K | 863 (19·29%) | 4,531 (20·33%) | 894 (19·14%) | 4,500 (20·37%) |
| 150K+ | 908 (20·30%) | 3,703 (16·62%) | 926 (19·82%) | 3,685 (16·68%) |
| (Missing) | 363 | 1,442 | 379 | 1,426 |
| Multimorbidity |  |  |  |  |
| 0 | 723 (15·41%) | 3,167 (13·70%) | 744 (15·19%) | 3,146 (13·74%) |
| 1 | 999 (21·30%) | 4,931 (21·34%) | 1,043 (21·29%) | 4,887 (21·34%) |
| 2 | 954 (20·34%) | 5,035 (21·79%) | 999 (20·39%) | 4,990 (21·79%) |
| 3+ | 2,015 (42·95%) | 9,979 (43·18%) | 2,113 (43·13%) | 9,881 (43·14%) |
| (Missing) | 146 | 616 | 152 | 610 |
| Pre-COVID physical activity |  |  |  |  |
| Below WHO threshold | 3,222 (67·45%) | 15,782 (66·94%) | 3,380 (67·76%) | 15,624 (66·87%) |
| Above WHO threshold | 1,555 (32·55%) | 7,795 (33·06%) | 1,608 (32·24%) | 7,742 (33·13%) |
| (Missing) | 60 | 151 | 63 | 148 |
| Pre-COVID social participation |  |  |  |  |
| Low | 2,528 (53·71%) | 12,590 (54·06%) | 2,704 (55·04%) | 12,414 (53·78%) |
| High | 2,179 (46·29%) | 10,698 (45·94%) | 2,209 (44·96%) | 10,668 (46·22%) |
| (Missing) | 130 | 440 | 138 | 432 |
| Pre-COVID mood disorder | 878 (18·72%) | 3,963 (16·99%) | 915 (18·67%) | 3,926 (16·99%) |
| (Missing) | 146 | 406 | 150 | 402 |
| Pre-COVID anxiety disorder | 448 (9·54%) | 2,055 (8·81%) | 476 (9·70%) | 2,027 (8·77%) |
| (Missing) | 141 | 403 | 145 | 399 |
| Baseline COVID anxiety screen, positive | 275 (7·10%) | 1,302 (5·92%) | 286 (7·07%) | 1,291 (5·91%) |
| (Missing) | 962 | 1,724 | 1,008 | 1,678 |
| Baseline COVID depression screen, positive | 1,068 (24·98%) | 4,770 (20·44%) | 1,116 (24·92%) | 4,722 (20·41%) |
| (Missing) | 561 | 386 | 573 | 374 |

*Notes*. COVID = coronavirus; WHO = World Health Organization.
